# Decentralizing treatment services with link ART centres- experience from Karnataka, South India

**DOI:** 10.1186/1742-4690-9-S1-P82

**Published:** 2012-05-25

**Authors:** Salma Fahim, Suresh Shastri, Bharat Rewari

**Affiliations:** 1 Karnataka State Aids Prevention Society, Bangalore, India; 2 National AIDS Control Organisation, India

## Background

Prior to 2007-08, ART services in Karnataka state, India were delivered through ART centres, located mainly in medical colleges, and tertiary and district hospitals. This led to high rates of defaults, increased travel time and cost and loss of working hours. To make treatment services more accessible to PLHIV, link ART centres (LACs) were developed at sub-district primary and secondary care levels, co-located with HIV voluntary counselling and testing centres.

## Methods

After a thorough needs assessment based on existing ART and HIV testing data which included load in ART centre, HIV prevalence, distance and accessibility, sites were identified, health personnel trained, and patients sensitized about the centres.

## Results

Currently there are 122 LACs attached to 29 nodal ART centres in the state. The LACs provide services to 5,498 PLHIV.Default rate has come down from 3.5 % in ART centres to less than 0.5% in LACs.The average patient travel distance has declined from 70 to 30 kms, saving travel cost and time. Waiting times for refilling prescriptions have declined from four hours to one hour, which has improved patient adherence. Health care staff at primary and secondary level care centres are gradually taking the lead in HIV care and treatment service delivery.

## Conclusions

The establishment of LACs at primary and secondary care levels has helped improve drug adherence and service access. They save travel cost and time, and more importantly, help strengthen primary and secondary health care services, which is beneficial for program sustainability.

